# A Review on Deterministic Lateral Displacement for Particle Separation and Detection

**DOI:** 10.1007/s40820-019-0308-7

**Published:** 2019-09-17

**Authors:** Thoriq Salafi, Yi Zhang, Yong Zhang

**Affiliations:** 1 NUS Graduate School for Integrative Sciences and Engineering, National University of Singapore, Singapore, 119077 Singapore; 2 Department of Biomedical Engineering, National University of Singapore, Singapore, 117583 Singapore

**Keywords:** Microfluidic, Deterministic lateral displacement, Particle separation, Particle detection

## Abstract

The separation and detection of particles in suspension are essential for a wide spectrum of applications including medical diagnostics. In this field, microfluidic deterministic lateral displacement (DLD) holds a promise due to the ability of continuous separation of particles by size, shape, deformability, and electrical properties with high resolution. DLD is a passive microfluidic separation technique that has been widely implemented for various bioparticle separations from blood cells to exosomes. DLD techniques have been previously reviewed in 2014. Since then, the field has matured as several physics of DLD have been updated, new phenomena have been discovered, and various designs have been presented to achieve a higher separation performance and throughput. Furthermore, some recent progress has shown new clinical applications and ability to use the DLD arrays as a platform for biomolecules detection. This review provides a thorough discussion on the recent progress in DLD with the topics based on the fundamental studies on DLD models and applications for particle separation and detection. Furthermore, current challenges and potential solutions of DLD are also discussed. We believe that a comprehensive understanding on DLD techniques could significantly contribute toward the advancements in the field for various applications. In particular, the rapid, low-cost, and high-throughput particle separation and detection with DLD have a tremendous impact for point-of-care diagnostics.

## Highlights

- A well-organized and thorough discussion on the fundamental principles and recent progress in deterministic lateral displacement (DLD) is provided.
- The most updated designs and applications of DLD techniques for particle separation and detection are reviewed.
- The current limitations of DLD and its potential solutions for clinical and commercial applications are discussed.

## Graphical abstract

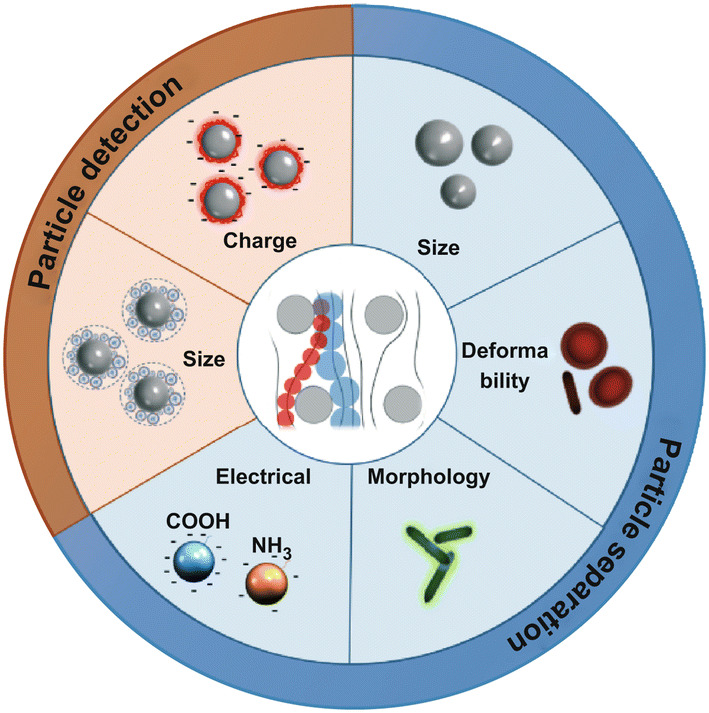

## Introduction

**Fig. 1:**
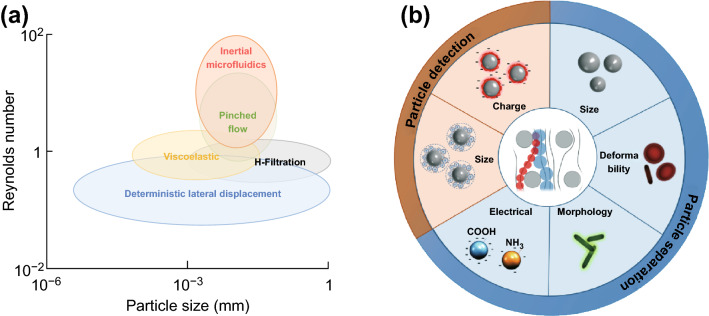
**a** Overview of the working range of passive microfluidics techniques. DLD mostly operates at a low Reynolds number and has high dynamic size separation range compared to other passive techniques. **b** A summary of DLD applications for particle separation and detection

The separation, isolation, and detection of particles in suspension are important for a wide spectrum of applications including biomedical research and clinical diagnostics. Typically, these tasks are performed by centrifugation, flow cytometry, gel electrophoresis, chromatography, etc. However, these techniques require a large volume of samples and have inevitable sample loss. Microfluidic has opened up the possibilities for miniaturizing these analytical devices through the precise control of small fluid volume on a microscale channel and has been widely used for particle manipulation such as focusing, fractionation, and sorting of micro- to nanoparticles [1]. These microfluidic platforms are able to provide portability, low cost, precise manipulation, and reduced sample volume for particle sorting. The microfluidic techniques for particle separation can be classified into active and passive types. The active microfluidic techniques manipulate the particles’ movement in a real-time manner by using external forces including magnetofluidics (magnetophoresis, dimagnetophoresis) [2], dielectrophoresis [3], acoustofluidics [4], and thermophoresis [5]. On the other hand, the passive microfluidics provide a simpler setup as they only require the intrinsic fluidic forces to drive the particle separation such as the hydrodynamic filtration [6], pinched flow fractionation [7], inertial microfluidics [8], viscoelastic separation [9], and deterministic lateral displacement (DLD) [10]. Among these techniques, DLD has been popular and widely used in the last decade for particle separation and detection. DLD is a robust passive microfluidic particle separation technique pioneered by Huang et al. in 2004 [11] to sort particles based on their size with pillar arrays. DLD holds a promise due to the low cost, robustness and provides a precise particle manipulation with a high-resolution separation. Compared to other passive microfluidic techniques for particle separation, DLD mostly operates at low Reynolds numbers and provides high dynamic size separation, which ranges from millimeter to micro- and nanometer sizes as seen in Fig. 1a.

For more than a decade, the theory of DLD has been studied and revised, new phenomena on DLD were discovered, and the various designs of DLD have been presented to achieve efficient and high-throughput particle sorting. Moreover, recent advances in DLD show its ability to sort particles based on their shapes, deformability, and electrical properties. Due to the high sensitivity of the separation with a resolution limit of 20 nm [11, 12], DLD has been widely used to sort, concentrate, and isolate many biological particles including circulating tumor cells [13], white blood cells [14], red blood cells (RBCs) [15], stem cells [16], parasites [17], spores [18], bacteria [19], exosomes [20], and DNA [21, 22]. Furthermore, recent progress enables the use of DLD pillar arrays as a platform for biomolecules detection including proteins and vesicles as seen in Fig. 1b [23].

Here, we will provide a comprehensive review on the fundamental and recent progress of DLD including the principle, device design, and factors influencing the critical diameter. Next, recent applications of DLD for particle separation and detection are presented. Lastly, the challenge of microfluidic DLD for various applications, its potential solutions, and the future directions are discussed.

## DLD Physics

### Fundamental of DLD

**Fig. 2:**
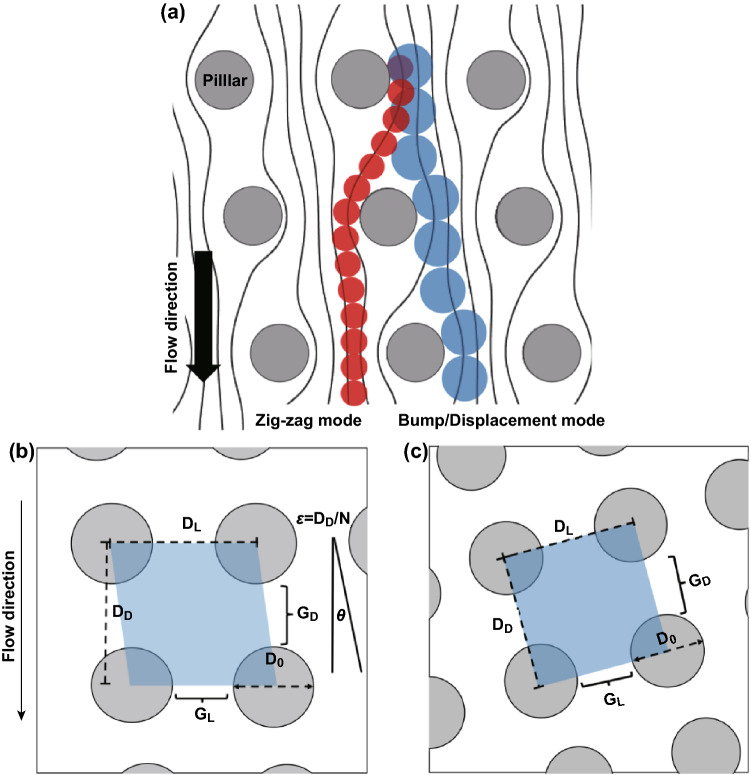
Fundamental of DLD. **a** Deterministic lateral displacement principle: small particles whose sizes are less than the critical diameter move in a zigzag direction, while large particles travel in bumping mode. **b** The parallelogram array layout design and **c** the rotated square array with the design parameters on each of the DLD unit cell. (*D*_L_, *D*_D_): center-to-center pillar distance in the lateral and downstream directions, *N*: array period, *θ*: the angle of the tilted array relative to the flow direction

Microfluidic DLD uses tilted pillar arrays that generate a fluid bifurcation and a unique number of streamlines between the gaps. Initially, Huang et al. [11] discovered that the total fluid flux on each gap can be divided to the periodicity (*N*) and suggested that the number of streamlines between each pillar corresponds to the periodicity of the DLD array. Particle flows in the DLD array are influenced by both the fluidic forces and the pillar obstacles effect. When the particle is located in the pillar gap, the particle with a radius smaller than the first width of the streamline will follow the initial streamline and travel in the zigzag mode, while the particle larger than the first streamline width will be bumped to the pillar and displace laterally to the next streamline as seen in Fig. 2a. The cutoff size parameter between the zigzag and displacement mode is known as the DLD critical diameter (*D*_c_). To develop the DLD array with a desired critical diameter size, there are multiple design parameters to be considered on the DLD unit cell, which comprise the lateral and downstream pillar gap (*G*_L_, *G*_D_), the row shift fraction ($\varepsilon$), and the pillar diameter (*D*_0_) as illustrated in Fig. 2b for the parallelogram array layout and Fig. 2c for the rotated array layout.

### DLD Critical Diameter Model

The understanding of the critical diameter is essential to design a DLD device for desired applications. Hence, several theoretical and empirical models to predict the critical diameter have been proposed. Inglis et al. [24] developed a theoretical critical diameter of the separation, which depends on two times the width of the first streamline, as shown in Eq. 1:

1 $$D_{\text{c}} = 2\beta$$

This model almost predicts the particle displacement between the zigzag and bumping mode, but it still underestimates the experimental critical diameter value from Davis et al. [25], who tested the particle separation behavior in many devices with different row shift fractions and gap sizes and proposed Eq. 2:

2 $$D_{\text{c}} = 1.4 G \varepsilon^{0.48}$$

where $\varepsilon = \tan \theta$ and $\theta$ denotes the tilted angle of the array with respect to the flow direction. This empirical formula has been popular for designing various DLD devices for different applications. However, this DLD model of binary separation is incomplete due to the existence of the intermediate displacement mode of separation. This intermediate displacement mode is caused by the uneven pressure between the lateral and downstream direction that disrupts the flow streamline, which is called as the anisotropic permeability effect [20, 26–29].

**Fig. 3:**
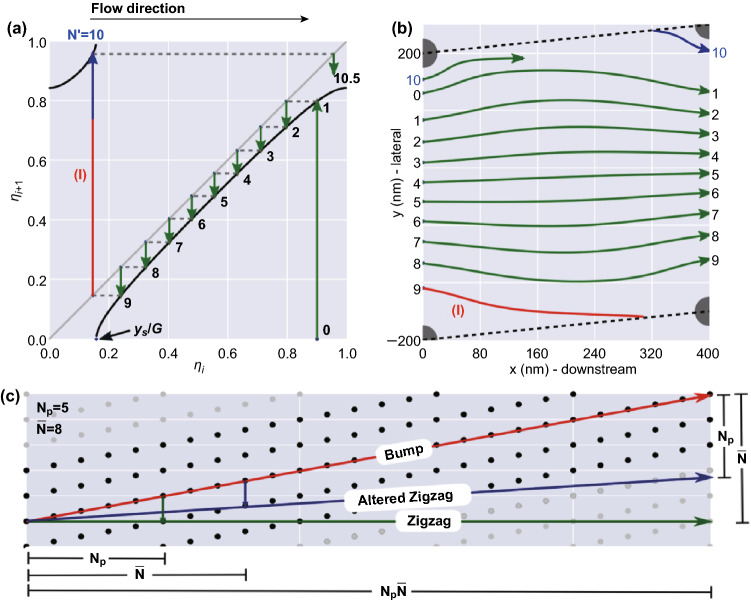
Unified DLD separation model. **a** The recurrence map of the particle trajectories generated from ten DLD unit cells along the array with *D*_0_ = 40 nm, *D*_L_ = *D*_D_ = 400 nm and *N*_p_ = 10 shows that the particle position at the end of the period is not the same as the initial position. The arrow represents the particle trajectory direction, the green line is the direct transition, the red line is the zigzag transition on the veering streamlines (*I*), and blue line depicts the particle enters the adjacent DLD cell unit. **b** The superimposed particle trajectories from ten DLD cells, which show the beginning and the ending positions of particles in each DLD cell. **c** Three different displacement modes of bump, altered zigzag, and zigzag from the unified DLD model. Figure panels reproduced from Ref. [30] with permission from the National Academy of Science, copyright 2017. (Color figure online)

Kim et al. [30] proposed a unified theoretical model that accounts for the intermediate displacement mode for small particles in a parallelogram DLD array. The model is based on the flow stream distortion due to the pillar array that leads to the pseudoperiodicity of the array that is different from its structural periodicity. This is unlike the previous DLD model, in which the symmetry of the fluid streamline follows the periodicity of the pillar array. A particle-tracking simulation study that plots the position of the particle in the DLD cells over the length of the array supports this notion [30]. Figure 3a, b shows the simulation of a single-particle trajectory over multiple consecutive DLD cells on a recurrence map. Initially, the particle travels from position 0 to position 1 in the next DLD cell and subsequently goes to position 2 and so on. The particle at position 9 corresponds to the veering transition through the zigzag trajectory mode to enter position 10 in the adjacent DLD cell. Due to the symmetry breaking of the fluid flow, the particle initial position (0th) is different from the tenth position, which results in the pseudoperiodicity of the array that is different from its structural periodicity as seen in Fig. 3c. The revised migration angle for this model is formulated as Eq. 3:

3 $$\theta = \tan^{ - 1} \left( {\frac{{\left( { \bar{N} - N_{\text{p}} } \right)D_{\text{D}} }}{{\bar{N}N_{\text{p}} D_{\text{L}} }}} \right)$$

where $\bar{N}$ is the pseudoperiodicity of the array obtained from the average of the local periodicity, *N*_p_ is the structural periodicity, *D*_L_ is the distance between pillars in the lateral direction, and *D*_D_ is the distance between pillars in the downstream direction. If the pseudoperiodicity is larger than the structural periodicity, the resulting zigzag migration angle is more than zero, which is called the altered zigzag mode that occurs solely due to the fluidic streamline effect and not the particle–pillar interaction. Furthermore, this model also explains the influence of the pillar diameter on the particle separation. All else equal, the increase in the ratio of the pillar diameter to the downstream distance (*D*_0_/*D*_D_) increases the local periodicity of each cycle that leads to a higher migration angle. The nanoparticle separation experiments conducted in the nanoscale DLD with various design parameters support this DLD theory [30–34].

### DLD Device Design for Binary and Multiple Separations

Microfluidic DLD can be designed with different device layouts and array arrangements for binary or multiple particle size separations, which have been implemented for different applications. Generally, this DLD device design can be classified to single or multiple critical diameter design.

#### Single Critical Diameter Design

**Fig. 4:**
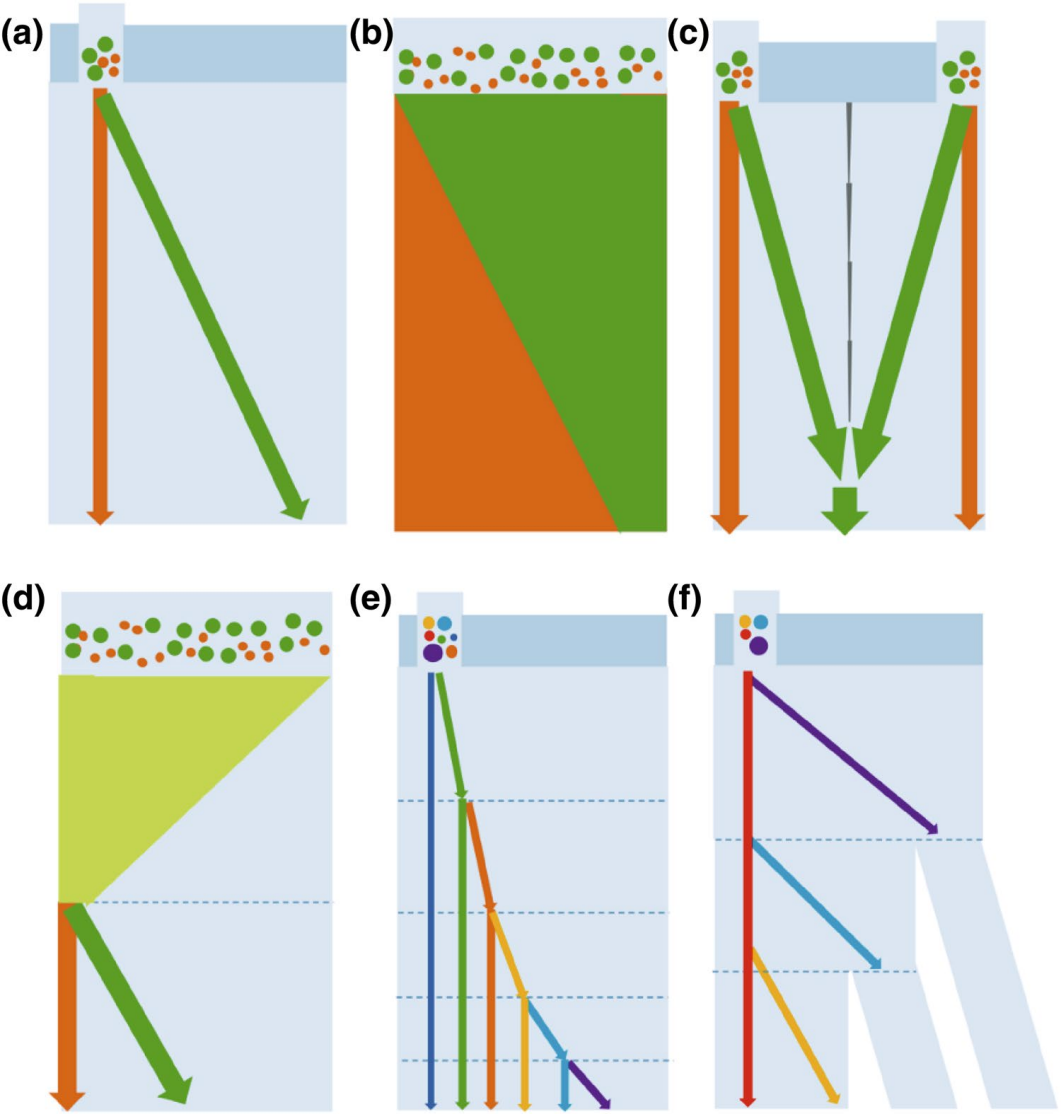
DLD device layout designs for binary and multiple separations. **a** Simple single array design, **b** condenser array design, **c** mirrored array design, **d** condenser and sorter array design, **e** chirped array design, and **f** cascade array design

The single critical diameter design separates particles in a binary fashion, which is based on a single uniform arrays design parameter along the entire channel. The layout for this DLD design typically requires three inlet reservoirs that comprise a sample inlet and two buffer inlets, with the sample inlet being sandwiched between the buffer inlets as seen in Fig. 4a. There is a simpler design with only one full-width sample inlet that concentrates the particle larger than the critical diameter, which is known as the condenser design (Fig. 4b). Although this design can achieve a high throughput rate, the purity of the sorted particles is less than the multiple inlet designs. Another type of common single *D*_c_ layout design is a mirrored concentrator array with one to three inlets. This device comprises two-mirrored pillar arrays that are typically separated by the center wall as seen in Fig. 4c. The particle larger than the critical diameter will be displaced and focused to the center, while the rest of the particles are flowing straight in the zigzag mode. Using the mirrored design, the particles are only required to be separated half of the device width as compared to the non-mirrored design, which infers that the length of the device can be reduced by half to provide a higher throughput rate [35].

#### Multiple Critical Diameter Design

The requirement of a specific gap size to generate a certain critical particle diameter suggests that in a single uniform array, a particle larger than the gap size will be trapped and eventually clog the device. To avoid this clogging, a multiple size separation with the DLD device can be applied through the condenser and sorter, chirped, or cascade post arrays design by varying the gap size or shift fraction parameters along the channel. Each design has a dynamic range characteristic, which is the ratio of the largest critical diameter which can be separated without clogging to smallest *D*_c_ within the device. The multiple critical diameter design comprises a condenser and sorter region, which is able to concentrate the polydisperse particles in the condenser region and subsequently separate them in the sorter design (Fig. 4d) [30]. The chirped device typically has the same gap size, but multiple row shift fractions along the channel that enable the continuous separation of polydisperse particles without clogging as seen in Fig. 4e. In the cascade array, the gap and the row shift fraction are varied along the channel and there are additional side channels to collect the separated particles from each of the critical diameter region (Fig. 4f). To design a cascade array, the resistance of the side channels must be comparable to the resistance of the main channel to provide the same flow rate for each channel. The cascade array can also be designed as a modular device that can be combined in a series or parallel manner for the separation of polydisperse particles [36, 37].

## Factors Influencing the DLD Critical Diameter

The control and tuning of critical diameter are essential to design DLD device for specific applications. There are several factors that can influence the DLD critical diameter including the device geometrical parameters, fluidic-related forces, particle-induced effect, surface interaction, and external forces.

### Geometry-Induced Effects

#### Pillar Shape Design

**Fig. 5:**
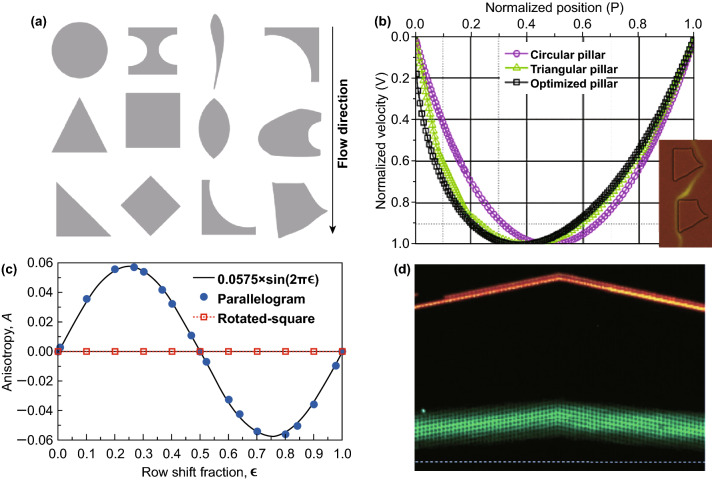
Geometry-induced effects on critical diameter. **a** Various DLD pillar shape designs. **b** The effect of the optimized pillar structure on the velocity flow profile that changes the DLD critical diameter [42]. **c** Comparison of the anisotropic permeability values for the parallelogram and the rotated square array [29]. **d** The deviation of the green beads displacement trajectory at the enabler region due to the anisotropic permeability of the parallelogram array [29]. Figure panels reproduced from Ref. [42] with permission from Elsevier, copyright 2017, Ref. [29] with permission from the Royal Society of Chemistry (CC BY 2017)

Since the critical diameter values depend on the fluidics streamline, several studies have investigated the effect of different pillar shapes on the fluid dynamics around the pillar as well as their critical diameters. The conventional circular pillar has been modified to different pillar shapes, including the airfoil shape [38], triangle [39], I-shaped [40], L-shaped and its variety [19], asymmetric shape [41], and optimized shape [42], which are then evaluated for their performance on the particle separation and the throughput as seen in Fig. 5a. For instance, Hyun et al. [42] showed the optimized pillar shape from the topological optimization that could reduce the critical diameter size as compared to the circular pillar due to the asymmetric velocity profile on the gap as seen in Fig. 5b. To predict the particle separation for different pillar shapes, Zhang et al. [43] developed a general equation for the critical diameter of different pillar shapes from the simulation of the circle-, square-, diamond-, and triangle-shaped pillar array:

4 $$D_{\text{c}} = \alpha G\varepsilon^{\beta }$$

in which $\alpha$ and $\beta$ are the dimensionless geometric coefficients for different pillar shapes [44]. The pillar shape affects not only the fluid streamlines but also the particle flow trajectory dynamics in DLD, especially for non-spherical and deformable particles. For instance, the airfoil-shaped pillar reduces the deformation of a non-spherical particle, which decreases its critical diameter and improves the performance of blood cells and trypanosomes separation [38]. Furthermore, the I-shaped pillar has both protrusions and grooves, which propels the red blood cells to rotate and displace laterally following the angle of the pillar [40].

#### Anisotropic Permeability of the Array

Microfluidic DLD can be designed with two different array layouts, which are the parallelogram and the rotated square array. Both array layouts are almost equally popular to be implemented, and the parallelogram array is slightly more preferred because it is relatively easier to design and implement for the cascaded/chirped array design. However, recent DLD studies show that the parallelogram array is prone to the anisotropic permeability due to the uneven pressure between the lateral and downstream directions that disrupts the flow streamline, while the rotated square array does not induce any anisotropic permeability as seen in Fig. 5c. This anisotropic permeability (AP) is defined by Eq. 5 [29]:

5 $${\text{AP }} = \frac{{\Delta P_{\text{lateral}} }}{{\Delta P_{\text{downstream}} }}$$

where $\Delta P_{\text{lateral}}$ and $\Delta P_{\text{downstream}}$ are the pressure gradients in the lateral and downstream directions, respectively. Moreover, in a DLD device design with an enabler, which is an interface gap between arrays that are typically present in a cascade array, the anisotropic permeability induces a secondary flow that generates a primary tilt of the fluid flow that is located away from the side walls. This results in the change in the critical diameter of the separation as seen in Fig. 5d. The aspect ratio of the downstream to the lateral gap and the gap to pillar diameter ratio also influence the magnitude of the anisotropic permeability.

The anisotropic permeability also depends on the row shift fraction ($\varepsilon$) value. It is shown that the DLD array whose row shift fraction value is less than 0.5 induces a positive anisotropic permeability, while DLD with the row shift fraction value of more than 0.5 results in a negative anisotropy [42]. Kim et al. demonstrated that the array pseudoperiodicity from the variation of lateral and downstream gap design is caused by the anisotropic permeability. They also showed a correlation between the normalized migration angle of the particle trajectories and the average flow velocity, which suggests that the effect of anisotropic permeability on particle separation can be estimated [30]. In addition, different pillar shapes have a different level of anisotropic permeability, with the highest anisotropy value observed in a highly asymmetric post shapes such as the right triangle pillar and I-shaped pillar [45]. Furthermore, the rotation of the pillar shape design is able to control the degree of the anisotropic permeability in the DLD array [33].

#### Sidewall Effect

In DLD arrays, the pillars are shifted in an angle on a straight channel, which leads the two sidewalls of the channel to intersect with the pillar arrays. This results in the disruption of the fluid flow on the channel wall side, which may result in the breakdown of the particle travel mode. To minimize the flow disruption caused by the edge wall, Inglis *et al.* proposed two equations to correct the edge streamlines by altering the design of the gap size at both ends of the row post [46]. For the DLD device with the direction of the pillar shift from the left to the right side, the gap between the sidewall and the side pillars can be calculated using Eq. 6:

6 $$G_{\text{left}} = G\sqrt {\frac{n}{N}} \,{\text{and}} \,G_{\text{right}} = G\sqrt {2 - \frac{n}{N}}$$

where $G_{\text{left}}$ and $G_{\text{right}}$ are the gaps on the left side and right side, respectively, *N* is the total number of rows, and n is the row number starting from 1 to *N*.

**Fig. 6:**
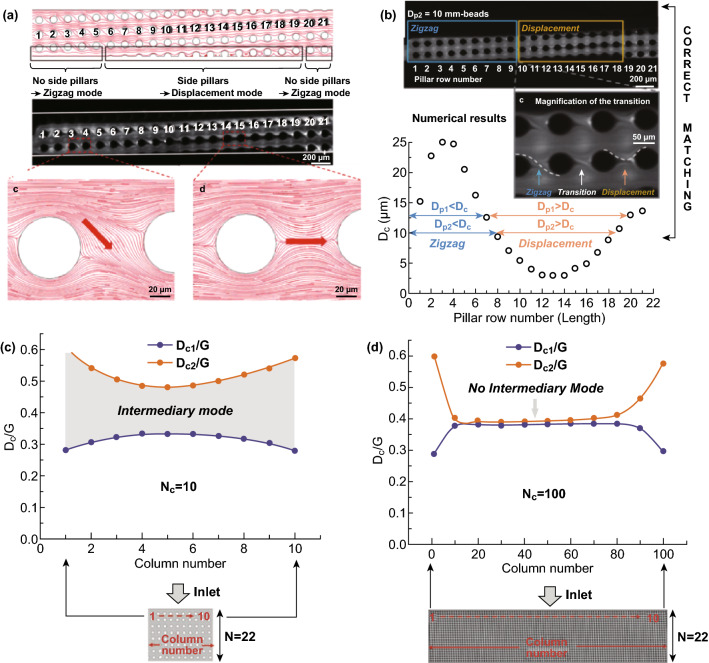
**a** Sidewall effect on the particle separation: The regions without the side pillar induce the zigzag transition, while the sidewall regions with the side pillars induce the displacement mode trajectory. **b** The variation in the critical diameter with the pillar row number shows a periodic critical diameter value trend with the highest peak of *D*_c1_ and lowest peak of *D*_c2_. **c** The value of *D*_c1_/*G* and *D*_c2_/*G* at different positions along the channel width in an array with *N* = 22 for ten pillar columns and **d** 100 pillar columns. Figure panels reproduced from Ref. [32] with permission from Wiley, copyright 2017

Without the sidewall correction, there exists an intermediate mode of particle trajectory displacement at the sidewall, which is comprised of the zigzag mode in the area next to the wall without the side pillars and gradually changes to a displacement mode at the area with the side pillars as seen in Fig. 6a. The recurring presence of the side pillars near the sidewall leads to the periodic variation in the cutoff diameter (*D*_c_) along the channel, with the observed highest peak of *D*_c1_ on the wall area without the side pillars and lowest peak of *D*_c2_ on the wall area with the side pillars as seen in Fig. 6b [32]. The periodic variation in the cutoff diameter and the extent of the intermediate displacement mode also depend on the number of pillars (*N*_c_) across the channel width. As the *N*_c_ increases, the periodic variation in the cutoff diameter is reduced and the values of *D*_c1_ and *D*_c2_ are getting similar, which result in the reduced intermediate mode as seen in Fig. 6c, d. Although the intermediate mode at a small channel width disrupts the binary DLD separation due to the wall effect, the theoretical model prediction of the intermediate modes may be employed as a design parameter to provide the avenue for multiple particle diameter separations, especially in narrow DLD devices, where the intermediary mode is maximized [32].

### Fluidic-Related Forces

#### Viscoelastic Effect

**Fig. 7:**
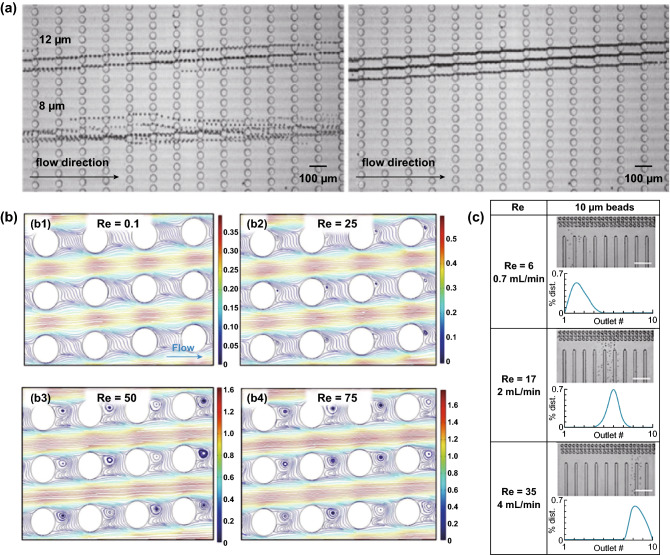
Modulation of the critical diameter using fluidic forces. **a** The modulation of critical diameters with viscoelastic fluids at a low fluid flow rate (left) and high flow rate (right) [48]. **b** The effect of high Reynolds numbers on the development of microvortices [49]. **c** The tuning of the critical diameter using different Reynolds number flows [49]. Figure panels reproduced from Ref. [48] with permission from Springer Nature (CC BY 2018), Ref. [49] with permission from Springer Nature, copyright 2018

Typically, DLD particle separation uses the Newtonian fluid as the buffer medium. The effect of a non-Newtonian fluid buffer medium on the DLD critical diameter has been studied theoretically, which suggests that the critical diameter can be tuned by using different polymer concentrations in the viscoelastic fluid buffer due to the fluid shear-thinning effect [47]. The change in the critical diameter is due to the modification of the parabolic profile on the pillar gap for the Newtonian fluid to a flatter profile for the non-Newtonian fluid, which reduces the first width streamline of the DLD array. Recently, the experimental validation on the effect of shear thinning using Xanthan gum solution (power-law fluid) confirmed this simulation study [48]. The elastic force of the fluid is also proven experimentally to influence the particle separation. Using the polyvinylpyrrolidone (PVP) solution (Boger-type fluid), the effect of the elastic force on the particle separation was investigated. It is shown that the critical diameter of the separation can be modulated by modifying the Weissenberg number (*Wi* = λ*u*/*D*_L_, *u* is the average fluid velocity and *λ* is the shear relaxation time). The application of high flow rate increases the particle effective diameter due to the effect of the normal stresses that manipulate the particle trajectory to focus on the center of the channel as seen in Fig. 7a.

#### Reynolds Number Effect

DLD is commonly operated at the Reynolds number of less than 1 with a flow rate in the µL min^−1^ range due to the high resistance of the pillar array. With the application of higher Reynolds number in DLD, the inertial forces become more dominant, which can change the fluidic streamline and eventually develop the microvortices behind the pillar structure [49]. The alteration of the streamlines and the presence of the microvortices at a high Reynolds number regime can modulate the DLD critical diameter due to the increase in the apparent diameter of the pillar [50]. The simulations with different Reynolds numbers in DLD pillar arrays show that the streamlines are still continuous at *Re* = 0.1, but start to form the microvortices behind the pillar structure at *Re* = 25, which fully mature at *Re* = 50. The microvortices then stop to develop after *Re* = 75 as seen in Fig. 7b. The microvortices size can grow to half of the pillar size, which disrupts the streamline surrounding the pillar structure. The experimental results with different flow rates and Reynolds numbers suggest that the increase in the Reynolds number can tune the critical diameter due to the alteration of the streamline, even before the formation of the microvortices as seen in Fig. 7c. The application of the airfoil-shaped pillar allows for the elimination of the microvortices in the high Reynolds numbers regime [51].

### Particle-Induced Effect

#### Particle Concentration

Particle concentration influences the particle separation in DLD because of the particle–particle interaction effect. Most of the reported DLD separation studies typically use a diluted sample concentration to deal with the particle crowding. This is because the non-dilute particle concentration may result in the disruption of the separation, which is mainly due to two reasons. First, the high particle concentration results in the particle collision that has a non-deterministic nature and the non-diluted concentration can also disrupt the streamline of the surrounding fluid, which changes the critical diameter of the separation [52, 53]. The immersed-boundary and lattice Boltzmann method (IB-LBM) fluid simulations for different red blood cell concentrations show a significant decrease in the displacement mode in a high hematocrit concentration as compared to lower hematocrit concentration [34, 52]. The effect of the high particle concentration has also been experimentally shown to reduce the separation efficiency as compared to a dilute particle concentration in a gravity-driven DLD [54]. Even though the disruption of the separation has been observed, more systematic and thorough experimental studies are required to understand the influence of non-dilute particle separations in DLD.

#### Particle Diffusion

**Fig. 8:**
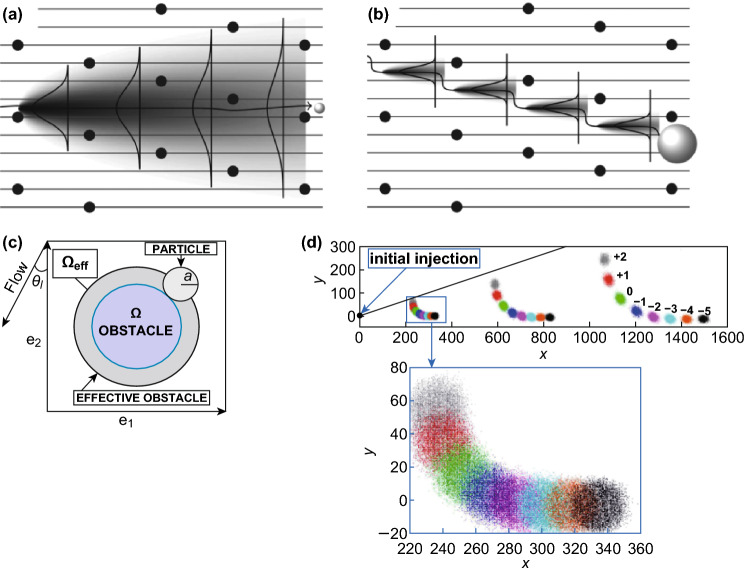
**a** Motion probability for small particles, indicated by the shaded region, is controlled by diffusion. An ideal position of a non-diffusive particle is indicated by the solid black trajectory straight line [59]. **b** Large particles have more dominated by the lateral displacement and are slightly affected by diffusion [59]. **c** Illustration of the effective obstacle concept. The blue area is the actual obstacle size, and the dark gray area represents the effective obstacle, which is the actual side of the particle [61]. **d** Chromatographic separation of particles with eight characteristic sizes in DLD under transient condition [55]. Figure panels reproduced from Ref. [59] with permission from IOP Publishing Ltd., copyright 2008, Ref. [61] with permission from Springer, copyright 2013, Ref. [55] with permission from Springer, copyright 2019. (Color figure online)

DLD is a passive microfluidic technique that does not rely on the external fields to overcome the diffusion of the particle. The presence of particle diffusion reduces the separation efficiency and precision, which is especially problematic for submicron particle separation [55]. The diffusion of particles in the microfluidic channel can be obtained using the Péclet number, which is the ratio of convection to diffusion [56]:

7 $$P_{\text{e}} = \frac{vL}{{D_{\text{f}} }}$$

where $v$ is the local speed of the fluid, *L* is the width of the channel, and $D_{\text{f}}$ is the diffusion coefficient of the particle, which depends on the size and shape of the particle. For spherical particles, the diffusion coefficient is

8 $$D_{\text{f}} = \frac{kT}{6\pi \mu a}$$

where *k* is the Boltzmann constant, *T* is the absolute temperature, $\mu$ is the dynamics viscosity of the fluid, and *a* is the hydrodynamic radius of the particle. Generally, the particles travel without a significant diffusion if $P_{\text{e}} \gg 1$ because the convection dominates the movement of the particles more than the diffusion [57]. Huang et al. showed that the diffusion effect influences the submicron particle separation in DLD, and this effect can be minimized by increasing the particle flow rate, which leads to a sharper transition from the zigzag to the bumping mode. A nanoscale DLD simulation study shows that at the Péclet number of 20, the diffusion is non-dominant and the DLD lateral displacement mode still occurs [58]. The experimental nanoparticle separation in a nanoscale DLD shows that the Péclet number of 4 is the limit of the breakdown for DLD as the diffusion is started to become more dominant compared to the convection of the fluid [20]. Several theoretical and experimental studies on the effect of diffusion and size dispersion on DLD pillar array have also been reported [59, 60]. Heller and Bruus proposed a discrete model that takes both diffusion and size dispersion effects into consideration [59]. The simulation of two particles with larger and smaller sizes characteristic shows that the smaller particles hardly interact with the arrays due to random Brownian motion, while the large particles are bumped on the pillars and slightly affected by the diffusion as seen in Fig. 8a, b [59].

The consideration of both deterministic motion and Brownian fluctuations at the microscale should not be ignored when dealing with the prediction of separation resolution. Cerbelli et al. [62] proposed a particle–obstacle interaction and advection–diffusion model for quantifying particle transport in a laminar flow through a periodic lattice of obstacles. The model focuses on the concept of effective obstacle as seen in Fig. 8c. The interaction between deterministic and stochastic components of particle motion leads to a new concept called enhanced effective dispersion regimes [61]. The degree of the dispersion enhancement is strongly influenced by the particle size and can yield an order of magnitude larger dispersion bandwidth [63]. They also designed a simple one-dimensional model, predicting the dispersion properties when a uniform force drags a diffusing tracer through a two-dimensional periodic assay. Experiments have shown that the interaction between the small-scale variations of the fluid velocity and the isotropic Brownian diffusion of the micrometric/nanometric particles gives rise to the convection-enhanced dispersion regimes, where the dispersion coefficient (*s*) can attain values that are orders of magnitude than the bare particle diffusivity [64]. By utilizing this interaction under transient condition, the separation based on the size is realized over time, which is also effective for particles with specific (critical) dimensions that are hardly distinguished in the steady-state separation process. This process mimics a classical chromatographic separation as illustrated in Fig. 8d [55].

#### Particle Shape and Deformability

The particle shape and deformability also determine the DLD separation performance since the critical diameter size of the non-spherical particle depends on its orientation in the lateral gap. This property is essential since biological particles range from spherical to non-spherical shapes as well as rigid to deformable membranes such as red blood cells and bacteria. Some bacteria have rod shapes, and the orientation of the bacteria in the pillar gap decides the trajectory in DLD arrays [40]. Similarly, the deformable red blood cells experience a high shear stress in the pillar gap and the cell apparent size could be reduced to 2 µm, which results in the poor lateral displacement. Furthermore, the deformable cell separation in DLD can be manipulated using the viscosity contrast effect by varying the ratio of the intracellular viscosity with the extracellular cell viscosity. The viscosity of the separation buffer influences the dynamics of the membrane deformation at different locations in the DLD pillar array. With different viscosity contrast ratios, the RBCs’ trajectory modes can be manipulated to show positive, neutral zigzag, and negative transport modes in DLD arrays [65].

### Surface Interaction Forces

**Fig. 9:**
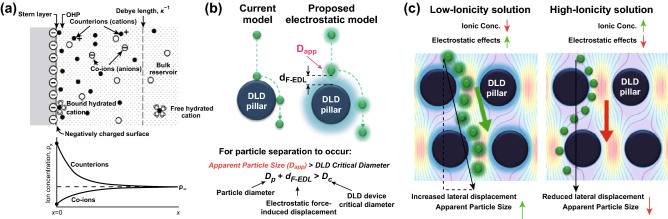
**a** Surface electrostatic ionic interactions in a charged wall [67]. **b** The proposed model of electrostatic double-layer force in DLD that modulates the apparent diameter of the particle [66]. **c** The electrostatic force enhances the particle lateral displacement at low ionic buffer strength, while the electrostatic charge is shielded at a high ionic concentration buffer [66]. Figure panels reproduced from Ref. [67] with permission from Elsevier, copyright 2011, Ref. [66] with permission from the Royal Society of Chemistry, copyright 2016

The separation of small particles in DLD is becoming more complex due to the emergence of the surface forces acting on the particles that can change the critical diameter [66]. Zeming et al. [66] discovered that the electrostatic force plays an important role in DLD as it can enhance and disrupt the sorting of the particle. Near the wall of a charged particle, there exists an electric double layer as the opposite counterions are attracted to the charged wall as seen in Fig. 9a. Due to this electric double layer, a charged particle will experience an electrostatic force when they are close to the charged wall. In DLD, the electrostatic force has been modeled to increase the actual particle diameter (*D*_p_) with the electric double-layer force length (*d*_F-EDL_) to become the apparent diameter *D*_app_ as depicted in Fig. 9b:

9 $$D_{\text{app}} = D_{\text{p}} + d_{\text{F {-} EDL}}$$

It has been shown that the DLD lateral displacement can be enhanced using the low ionic strength due to the larger electrostatic repulsion between the particle and pillars, which leads to a larger apparent diameter, while the lateral displacement is reduced at a high ionic concentration buffer due to the electrostatic shielding as seen in Fig. 9c. Using the ultra-pure deionized water as the buffer, the nanoparticle separation of 50 nm from 190 nm in 2-µm-gap DLD has been demonstrated [66]. A force equation model (Eq. 10) has been proposed to predict the particle apparent diameter by equating the drag force with the electrostatic force, which results in the *d*_F-EDL_ as seen in Fig. 9b:

10 $$d_{\text{F {-} EDL}} = - \lambda_{\text{D}} \ln \left[ {\frac{{ - \sigma_{\text{p}} \sigma_{\text{s}} + \sqrt {\sigma_{\text{p}}^{2} \sigma_{\text{s}}^{2} + \frac{{\left( {\sigma_{\text{p}}^{2} + \sigma_{\text{s}}^{2} } \right)3\mu \varepsilon_{\text{o}} \varepsilon_{\text{r}} V_{\text{bulk}} }}{{\lambda_{D} }}} }}{{\sigma_{\text{p}}^{2} + \sigma_{\text{s}}^{2} }}} \right]$$

where $\lambda_{\text{D}}$ is the Debye length, $\varepsilon_{\text{r}}$ and $\varepsilon_{\text{o}}$ are the electrical permittivity of the medium and free space, respectively, $\sigma_{\text{p}}$ and $\sigma_{\text{s}}$ are the surface charge density of the particle and the pillar surface, respectively, $\mu$ is the viscosity of the fluid, and *V*_bulk_ is the relative velocity of the surrounding fluid. Based on this formula, it has been shown that the particle apparent diameter is enhanced for more than 150 nm on the oxygen plasma-activated PDMS surface as compared to the native PDMS due to the stronger electrostatic force in a highly negative charge of SiO^−^ on the plasma-activated PDMS device [23]. Furthermore, the use of alkaline pH buffer using the NaOH solution improves the apparent diameter of the separation since the alkaline buffer ionizes the SiOH surface group to increase the negative surface charge group on the PDMS surface. Similarly, the particles with different surface charges and functional groups, including the plain polystyrene (PS) beads, PS-COOH, and PS-NH_3_, are shown to have a different apparent diameter of the separation in DLD, even though the actual sizes of the beads are the same.

### External Forces

#### Mechanical Stretching

**Fig. 10:**
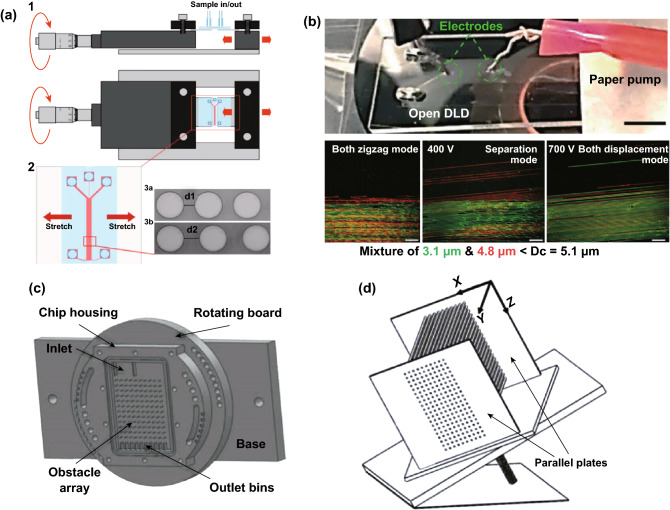
Modulation of the critical diameter with external forces. **a** Tuning of critical diameter using elastomeric stretching to increase the gap size and change the critical diameter [68]. **b** Active tuning of the critical diameter using dielectrophoresis force [69]. **c** Centrifugal-driven DLD for modulation of forcing angles [70]. **d** Gravity-driven DLD for modulation of forcing angles [54]. Figure panels reproduced from Ref. [68] with permission from the Royal Society of Chemistry, copyright 2008, Ref. [69] with permission from the Royal Society of Chemistry (CC BY 2017), Ref. [70] with permission from Springer Nature, copyright 2016, Ref. [54] with permission from Springer Nature (CC BY 2016)

The first report on the modulation of the critical diameter using external forces was achieved by using the elastomeric stretching of the PDMS-based DLD device by a micrometer precision screw [68]. The mechanical pulling force on the PDMS stretches the pillar array that leads to a larger gap size of the array and results in the increase in the critical diameter value as seen in Fig. 10a. With this method, the modulation of critical diameters from 7 µm to more than 9 µm depending on the extent of the PDMS stretching is reported. Although this method could provide a high degree of dynamic range by stretching the gap size, it requires a precise force stretching equipment to produce accurate control for modulating the gap size deformation. Furthermore, the stretching system can only increase the gap size and hence the critical diameter, while the elastomeric compression for reducing the gap size is more difficult to perform.

#### Dielectrophoresis Force

Another method that has been proposed to change the critical diameter is using AC external electric fields to generate an insulator-based dielectrophoresis (iDEP) force. This iDEP is based on the electric field deformation by the pillar array between the electrodes located at both ends of the channel (parallel direction) or across the channel width (orthogonal direction) [71]. The non-uniform electric field in the pillar array produces a DEP force that leads to the movement of the particles toward the center of the gap, which increases the particle lateral displacement. Beech et al. [12] reported the simulation and experimental validation about the critical diameter modulation using DEP and showed the critical diameter tuning from 2 to 6 µm in a single device by changing the voltage and frequency of the external electric field parallel to the array direction. The modulation of the critical diameter using the DEP force parallel to the direction of the array is also demonstrated on the open-channel DLD array as seen in Fig. 10b [69]. In another report, the AC electrokinetic DLD is applied in the orthogonal direction of the array to tune the critical diameter of the separation [72]. The method can deflect particles with the size of 3 and 1 µm in a DLD array with the critical diameter of 6.3 µm through a combination of electrophoresis, electroosmosis, and dielectrophoresis force based on the different applied electric field frequencies and electrolyte conductivities. At the low frequencies of the electric field (< 500 Hz), the particles experience the oscillation movement in the direction of the electric field, which is caused by the electrophoresis/electroosmosis (EP/EO) forces. This oscillation results in the increase in the effective particle diameter that leads to the displacement mode trajectory. As the frequency of the electric field increases, the amplitude of these oscillations is minimized and the dielectrophoresis (DEP) becomes the dominant electrokinetic force on the particles. At the high frequency of AC electric fields and sufficiently high electrolyte conductivity, the experiments show that the particles undergo negative DEP and are separated based on their effective particle diameters. Meanwhile, the particles are separated based on their electrical polarizability at the high frequency of electric field and low electrolyte conductivities [72]. In a recent work, Beech et al. [73] demonstrated the DLD device combined with the electrodes directly integrated onto the posts through the metal coating on the post to generate DEP force that is able to tune the critical diameter by a factor of 24 × (from *D*_c_ of 6 µm to 250 nm). Although this method provides a high dynamic range of the critical diameter tuning and improves the separation throughput due to the larger pillar gap, it requires additional external electrodes and AC electric field source with a high electric field strength that increases the complexity of the DLD experimental setup.

#### Driving Force Angle

The modulation of the critical diameter in DLD can also be performed with the external driving force that can control the forcing angle of the particle, virtually changing the row shift fraction of the pillar arrays. Several different external fields including gravity, centrifugation, and electrokinetic forces have been employed to change the critical diameter through the modulation of the forcing angle as seen in Fig. 10c, d [54, 70, 74]. In the gravity-driven DLD, the change in the orientation angle of the pillar array has been demonstrated to tune the critical diameter of the separation. The gravity-driven 3D-DLD that uses the cylindrical pillar has also been reported to separate particles with polydisperse sizes [54]. Similarly, the modulation of forcing angles using the centrifugal force has been reported to control the dynamic of the separation in DLD by rotating the chip holder on the centrifugation machine to change the critical diameter of the separation [70]. The modulation of critical diameter using the forcing angle has also been demonstrated in the electrokinetically driven DLD with the electric fields to drive the electroosmosis flow. By changing the orientation angle of the electric fields, the electroosmosis flow forcing angle can be controlled to produce the desired critical diameter for particle separations [74].

## DLD Applications

With the development of DLD technology, there are multiple modes of DLD applications that are mostly demonstrated for medical and biological research applications, including the particle separation, concentration, buffer exchange, and label-free detection. Initially, DLD uses the particle size parameter for the basis of the separation. Recent progress in DLD shows that several additional particle properties including shape, deformability, and electrical property can be used as the separation parameters. The recent applications of DLD and their corresponding designs and setups are summarized in Table 1.

**Table 1:** DLD applications by the separation parameter reported from 2014 onward

Samples	Pillar shape	Critical diameter	Parameters	Device material and fabrication	Device pre-treatment	Buffer	Flow rate	Recovery rate, purity, remarks	Refs.
*Size-based separation*									
Size-based separation of microparticles									
Separation of viable and non-viable mammalian cells	Circular	6.6 μm	*D*_post_ = 60 μm, *ε* = 0.05, and *G* = 20 μm, *H* = 40 μm	PDMS bonded to glass made from soft lithography from SU-8 mold	PBS solution 1% (w/v) BSA for over 60 min	1% PBS (w/v) bovine serum albumin	1.2 mL/h	Viable cells at 100% capture efficiency, at a purity of 23.1%	[75]
Separation of MCF-7 cells from RBCs	Circular	5.1 μm	*D*_post_ = 30 μm, G = 11 μm gap, depth = 24 μm	Open-channel DLD based on PDMS from soft lithography from SU-8 mold	Immersing the device in aqueous buffer solution	AutoMACS® buffer (isotonic PBS solution containing 2 mM EDTA, 0.5% BSA and 0.09% azide)	71 ± 19 nL/s	95% of cells recovered using membrane paper pump	[69]
Main and satellite droplet separation	Circular	37.1 μm	*D*_post_ = 100 μm, *ε* = 0.1, and *G* = 80 μm	PDMS bonded to glass made from soft lithography from SU-8 mold	Layer-by-layer (LbL), three layers of 0.1% poly (allylamine hydrochloride) and 0.1% poly (sodium 4-styrenesulfonate)	DI water	3 mL/h	100% purity and recovery efficiency	[76]
Separation of white blood cells from whole blood	Circular	8 μm, 5 μm, 4 μm	*D*_post_ = 4 μm, *G* = 18 μm, and *D*_post_ = 16 μm *G* = 12 μm, and *D*_post_ = 10 μm *G* = 9 μm	Plastic chips embossed from the soft elastomeric mold	Primed with PBS, 5 mM EDTA and either 1% BSA or 1% poloxamer	PBS containing 1% BSA	10 μL/min	Recovered 88% WBCs and removed > 99% RBCs and > 99% of unbound mAb	[14]
Separation of white blood cells from apheresis sample	Diamond	4 μm	*G* = 16 µm, and *ε* = 1/42	Plastic chips embossed from the soft elastomeric mold	Primed with PBS, 5 mM EDTA and 1% BSA	PBS containing 1% BSA	~ 70 mL/h	80% cell recovery, 87% platelet depletion	[77]
Microvesicle separation	Circular	250 nm	*G* = 6 μm, *θ* = 0.16°	PDMS bonded on silicon wafer	1 mg/mL PEG in 95% ethanol, 5% deionized water, and 3% (m/v) BSA in PBS	3% PBS buffer	3.74 mL/h	Recovery efficiency of 39% with purity of 98.5%	[57]
Red blood cells separation	Circular	2 to 3 μm	*G*_L_ = 9, *G*_D_ = 4 μm, *D*_Post_ = 9 μm, *θ* = 2.5°, *h* = 10 μm	PDMS bonded on glass made from soft lithography from SU-8 mold	1% Pluronic-treated PDMS surface	1 × PBS buffer	0.5 μL/min	91.2% separation index	[31]
Separation of PC3 prostate cancer cells from blood	Right isosceles triangles	6 μm	60 μm right isosceles triangles, *G* = 40 μm row shift = 1/50, *H* = 160 μm	Etched silicon device covered by a sealing tape of thin silicone layer with a polyolefin backing	2 mg/mL Pluronic F-108	PBS, 1% BSA, 5 mM EDTA, and 40 μM PPACK	14 mL of blood, 20 cm/s	86% cell collection yield	[78]
Separation of circulating tumor cells from blood	Circular	6 μm	*D*_post_ = 15 μm, gap = 15 μm, shift = 3 μm	Etched silicon device sealed by thin glass cover	PBS buffer containing 0.5% FCS and 0.1% sodium azide	PBS buffer containing 0.5% FCS and 0.1% sodium azide	Sample inlet = 7 μL/min	CTC enrichment from 0.0076 to 88%	[13]
Circulating tumor cells cluster separation from blood	Circular and asymmetric pillar	30 μm	1st stage: *D*_post_ = 50 μm, *G* = 63 μm, $\varepsilon$ = 1/7 2nd stage: *D*_post_ = 77 × 60 μm, *G* = 63 μm, $\varepsilon$ = 1/7	PDMS bonded on glass made from soft lithography from SU-8 mold	Primed with 70% (v/v) ethanol in water, flushed with 300 µL PBS, blocked with 3% Pluronic F-68 (w∕v) in PBS for 45 min	PBS buffer	Sample inlet 8.3 µL/min	99% recovery of large clusters, over 87% cell viabilities	[41]
Size-based separation of nanoparticle									
Separation of 50 nm and 100 nm particle	Circular	80 nm	*D*_D_/*D*_L_ of 1 and 2, and *D*_post_ = *G* = 200 nm, and *D*_L_ = 400 nm, *θ* = 2.86°	Optical contact lithography and a reactive-ion etch and a combination of electron-beam and deep-ultraviolet lithography	Capillary wetting of 2% Tween 20 in deionized water	Tween 20	300 µm/s	Up to 100% separation	[30]
Separation of 0.02 to 0.11 μm bead and exosomes	Circular	20–110 nm	*G* = 42, 118, 134, 214, 235 nm, *D*_post_ = 375 nm *θ* = 5.7◦	Optical contact lithography and a reactive-ion etching and a combination of electron-beam and deep-ultraviolet lithography	10 mM solution of dimethylchlorosilane in anhydrous chloroform was added to the reactor	diH_2_O for beads, PBS for exosome	0.1–0.2 nL/min (200–300 µm/s)	Up to 100% separation	[20]
Genomic length T4 DNA separation	Circular	0.7 μm	*G* = 1.7 μm, *D*_post_ = 6.3 μm, *θ* = 3.8◦	Etched silicon devices by reactive-ion etching	Primed with PEG	0 to 10% PEG buffer	0.0625–0.625 µL/h	Using PEG depleted source, 87-fold concentration	[21]
Exosomes isolation from serum and urine sample	Circular	95 nm	*G* = 225 nm, *D*_post_ = 175 nm nm, $\varepsilon$ = 0.1	Multi steps: array definition, bus patterning, glass bonding, silicon polishing, and etching	5% w/v BSA in a 1 × phosphate buffer saline	1 × PBS	Up to 900 μL/h	Integrate 1024 nano-DLD devices, up to 60-fold concentration	[79]
Separation of dsDNA with size of 100 bp	Circular	31–120 nm	*G* = 250 nm, *D*_post_ = 78–750 nm, $\varepsilon$ = 0.1	Optical contact lithography and a reactive-ion etching and a combination of electron-beam and deep-ultraviolet lithography	1 × TE buffer (10 mM Tris, 1 mM EDTA, pH 8), 3% mercaptoethanol, 0.1% TWEEN 20	1 × TE buffer (10 mM Tris, 1 mM EDTA, pH 8)	200 µm/s	75% recovery and threefold concentration of dsDNA sample	[22]
Morphology/shape-based separation									
Different shape particles (cubes, cylinder, tetrahedron, sphere, pyramids)	Circular	Varies with driving angle	*D*_post_ = 1 mm, *G* = 5 mm, *θ* = 15.8° to 32.0°	3D printed lattice pillar array	NA	DI water	Gravity-driven DLD	Uses 3D-DLD system	[80]
Separation of bacteria by chain length	Circular	1.24 μm	*D*_post_ = 12 μm, *G* = 4 μm, *θ* = 2.50° Depth = 9.8 μm	PDMS bonded to a thin layer of PDMS on glass slide	Treated with 0.2% (w/v) PLL (20)-g [3.5]-PEG for 20 min	PBS + 1% BSA solution	1.0 μL/h	100% single and chain bacterial	[81]
Red blood cells separation	I-shape, L-shape/inverted L-shape	3.32 μm	*G* = 10 μm and *ε* = 1/20	Silicon device from reactive-ion etching sealed with PDMS	Surface coating of trichlorosilane and treatment of Pluronic F-127 solution	PBS buffer diluted 20 ×	0.2 μL/min	100% separation	[19]
Separation of trypanosome from RBCs	Circular	5 μm	*D*_post_ = 20 μm, *G* = 12 μm, depth = 9 μm	Open-channel DLD, based on PDMS from soft lithography from SU-8 mold	Immersing the device in aqueous buffer solution	AutoMACS^®^ buffer (isotonic PBS solution containing 2 mM EDTA, 0.5% BSA and 0.09% azide)	71 ± 19 nL/s	Uses shallow depth, driven by paper pump	[69]
Separation of trypanosome from whole blood	Circular	7.1 μm, 3.5 μm, 3.5 μm	*D*_post_ = 20 μm, *G* = 22 μm, Depth = 26 μm *ε* = 1/21; *G* = 12 μm, Depth = 3.5 μm, *ε* = 1/26; *G* = 12 μm, Depth = 9 μm, *ε* = 1/26	PDMS devices based on SU-8 mold from three separate cycles of UV-lithography	Primed with 0.2% (w/v) PLL (20)-g [3.5]-PEG (2) in deionized water and treated with 20% fetal calf and 2 mM EDTA	AutoMACS^®^ buffer (isotonic PBS solution containing 2 mM EDTA, 0.5% BSA and 0.09% azide)	0.25 to 3.8 μL/min	Three different depths in series, driven by a simple pressure pump based on the disposable syringe and valve	[17]
Deformability-based separation									
Red blood cells separation	Circular	1.43 to 4.52 μm	*D*_post_ = 20 µm, *G* = 12 µm, *ε* = 0.025 to 0.275, *H* = 11 μm/*H* = 4 μm	PDMS bonded on glass made from soft lithography from SU-8 mold	0.2% PLL (20)-g [3.5]-PEG and rinsed after 20 min with autoMACS	AutoMACS^®^ for viscosity contrast *C* = 5, Dextran-500 11%, 4.5%, and 1% for *C* = 0.25, 1, and 2	0.5 mm/s in thick device, 0.12 mm/s in thin devices	Up to 100% separation of RBCs using the viscosity contrast	[65]
Separation of stiff and deformable red blood cells	Circular	3 to 9 μm	*D*_post_ = 20 μm, and *G* = 12 μm, chirped ε	PDMS bonded to glass made from soft lithography from silicon master	Primed with a 0.5% (w/v) of Pluronic F-108 in PBS for 20 min	PBS with 0.1% w/v Pluronic F108	Pressure between 0 and 1300 mbar	Up to 100% separation between deformable and stiff RBCs	[82]
Circulating tumor cells separation	Triangle	6 μm	*D*_post_ = 30 μm side length, 30 μm height, and Gap = 25 μm	PDMS bonded on glass made from soft lithography from SU-8 mold	Treated with 5% Kolliphor P-188 to block nonspecific adsorption	1 × PBS buffer	12 mL/h	90% capture yield, and 80% capture purity	[83]
Enrichment of human skeletal stem cell from bone marrow	Circular	7.5 μm	*D*_post_ = 29.6 μm, gap = 26.4 μm, *θ* = 2.05°	Device bonded on PDMS-coated glass slides made from soft lithography from SU-8 mold	Primed with 0.5% Pluronic F-127 in PBS 1 ×	BSA and 2 mM EDTA 1 × PBS buffer	15 to 30 μL/min	Separation based on the size and deformability of the cells	[16]
Electrical properties-based separation									
Separation of 50 nm to 1 μm beads particle	Circular	350 to 1000 nm	*D*_post_ = 6 μm, *G* = 2 μm, 14 section of *ε* = 0.013 to 0.117	Device made by dry etching of silicon wafer	1% Pluronic-treated PDMS surface	Low ionic to high ionic concentration of NaCl solution	0.05 μL/min	Using electrostatic effect	[66]
Separation of PS-NH_3_ and PS-COOH polystyrene beads	Circular	700 to 2000 nm	*D*_post_ = 6 μm, *G* = 4 μm, 14 section of *ε* = 0.013 to 0.117	PDMS bonded on glass made from soft lithography from SU-8 mold	Plasma-treated surface of PDMS	Different concentrations of NaCl buffer	0.1 μL/min	Using electrostatic effect	[23]
DLD for particle detection									
Protein detection (via coating on microbeads)	Circular	350 to 1000 nm	*D*_post_ = 6 μm, *G* = 2 μm, 14 section of *ε* = 0.013 to 0.117	PDMS bonded on glass made from soft lithography from SU-8 mold	Plasma-treated surface of PDMS	Different concentrations of NaOH buffer	0.1 μL/min	Limit of detection = 150 pM	[23]
Vesicle detection (via coating on microbeads)	Circular	350 to 1000 nm	*D*_post_ = 6 μm, *G* = 2 μm, 14 section of *ε* = 0.013 to 0.117	PDMS bonded on glass made from soft lithography from SU-8 mold	1% Pluronic-treated PDMS surface	0.1 × PBS buffer	0.1 μL/min	Limit of detection = 3.75 μg/mL	[23]

### DLD for Particle Separation

#### Applications of Size-Based Separation

In the size-based separation, the particles with different sizes have different trajectories and migration angles. This separation mode is the most widely implemented method and has been used for the separation and concentration of micro- to nanoparticles, including synthetic beads, droplets, and various biological samples like blood cells, bacteria, exosomes. Other modes of applications that rely on the size-based separation such as buffer exchange and on-chip washing have also been reported.

##### Size-Based Separation of Microparticles

**Fig. 11:**
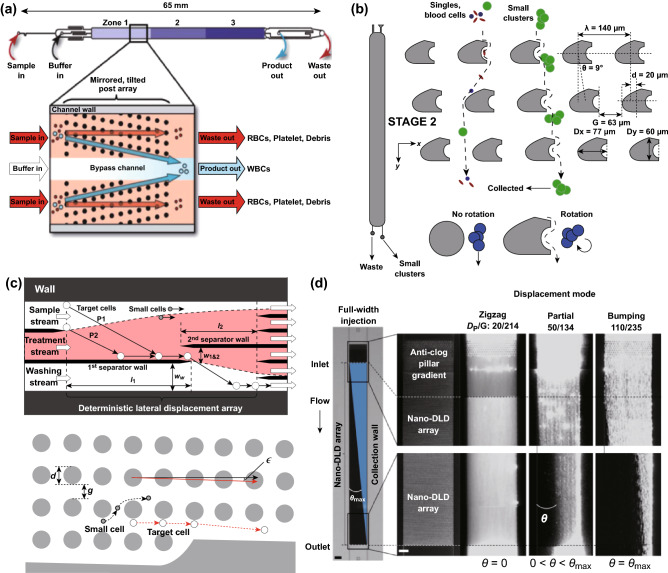
Applications of size-based DLD separation. **a** The design of the microfluidic DLD mirrored array for automated leukocyte processing [84]. **b** The two-stage DLD arrangement based on both the size and asymmetry for CTC clusters separation [41]. **c** The on-chip microfluidic DLD for chemical treatment and washing [85]. **d** The condenser array in a nano-DLD shows the zigzag, intermediate, and bumping modes for different nanoparticle diameters and gap sizes [20]. Figure panels reproduced from Ref. [84] with permission from Wiley, copyright 2016, Ref. [41] with permission from Springer Nature (CC BY 2017), Ref. [85] with permission from AIP Publishing, copyright 2015, Ref. [20] with permission from Springer Nature, copyright 2016

One famous contribution of DLD separation is in the development of hematology tools. DLD brings the complex and expensive fractionation tools for blood cells to an affordable and efficient on-chip separation. For example, inexpensive plastic DLD microchips can directly separate leukocytes without any other manual handling of samples with a recovery rate of 88% of input leukocytes as seen in Fig. 11a [84]. In another work, DLD mirrored arrays incorporating the diamond-shape pillars are used to process apheresis blood products, with 80% of the cell recovery rate and 87% of platelet depletion. Furthermore, DLD isolation of T cell converts more of the cells to the T-central memory phenotype with less variation as compared to the Ficoll-Hypaque (Ficoll) and direct magnetic methods, which benefits the downstream cell processing to manufacture the therapeutic cells [77].

Besides the blood cells separation, DLD technology has a great contribution in cancer research, especially for the isolation of circulating tumor cells (CTC) [86]. Au et al. [41] presented a two-stage DLD separation array based on the size and asymmetry to isolate CTC clusters with 99% recovery, 87% cell viabilities rate, and a minimal cluster dissociation as seen in Fig. 11b. In addition to the particle fractionation, the DLD size-based separation can be used for buffer exchange and labeling. Chen et al. proposed a wall-separated DLD device for on-chip leukocyte staining with Rhodamine 6G and downstream washing, without any manual handling or extra processing steps as seen in Fig. 11c. The ‘wall-separated’ design greatly improves the treatment time and decreases the chances of contamination. This design can also be applicable for on-chip processing like labeling and fixing [85].

##### Size-Based Separation of Nanoparticles

DLD has been popular for separation of microparticles, but there are only a few reports available for the nanoparticle separation. The separation of DNA, protein, and exosomes is currently still challenging due to the nanoscale size of the molecules. With a microfabricated DLD array, Santana et al. [57] designed a DLD array to separate microvesicles from populations of cancer cell-derived extracellular shed vesicles based on their different sizes. Another research also employed the DLD principle to separate microvesicles from serological samples from red blood cells and peripheral blood mononuclear cells [87]. On the other hand, the separation of biomolecules such as DNA molecules with a microfabricated array can be achieved only by deploying the external forces or chemical treatments to the sample. For instance, Huang et al. [11] used electric-driven DLD to separate artificial bacterial chromosomes, while Chen et al. [21] compacted genomic DNA molecules with polyethylene glycol (PEG) to minimize the coil size and increase the shear modulus of DNA globules in a DLD array to isolate the genomic DNA.

With the advances in the nanofabrication, DLD now can sort and enrich nanoparticles precisely and efficiently based on their size. Wunsch et al. [20] translated the DLD to a true nanoscale with their nanofabrication method to produce DLD arrays with the gap sizes from 25 to 235 nm, which can sort nanoparticles and exosomes whose sizes are between 20 and 110 nm as seen in Fig. 11d. Furthermore, the nanoscale DLD has been employed to separate dsDNA ranging from 100 to 10,000 base pairs (bp) with the 200 bp resolution [22]. In the nanoscale DLD, the particle sorted is smaller in size and the flow rate applied is lower due to the high resistance of the channel, which increases the particle diffusion effect. It has been shown that there is only 32% of particle lateral displacement for 20-nm particle at 42 nm gap size as the diffusion starts to overcome the deterministic process in nanoscale DLD. Although the nanoscale DLD is able to achieve the particle separation down to 20 nm in size, the nanofabrication is complex and has very low separation throughput, which is in the range of 0.1 to 0.2 nL min^−1^.

#### Applications of Morphology/Shape-Based Separation

**Fig. 12:**
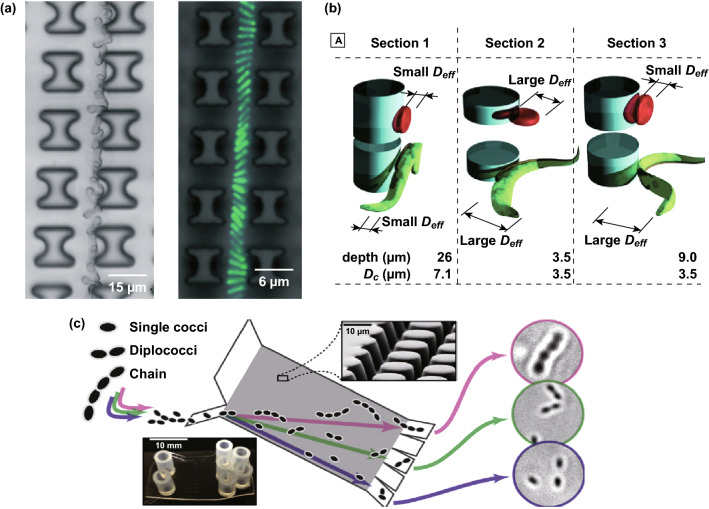
Applications of morphology-based DLD separation. **a** The separation of red blood cells and rod-shaped bacteria in I-shaped DLD [19]. **b** The separation of red blood cells and trypanosomes based on the shape with the use of different device depths in a series configuration [17]. **c** The separation of bacterial pathogens based on the different bacterial chain lengths [81]. Figure panels reproduced from Ref. [19] with permission from the Royal Society of Chemistry, copyright 2014, Ref. [17] with permission from the Royal Society of Chemistry, copyright 2016, Ref. [81] with permission from Elsevier, copyright 2018. (Color figure online)

The shape or morphology of particles influences the particle trajectory in DLD due to the dynamic of the particle orientation on the pillar array. Jiang et al. [80] reported the shape-based separation with the gravity-driven DLD to separate particles with different geometrical shapes including cubes, cylinder, tetrahedron, sphere, pyramids. The ability to separate particles by morphology is essential as some bioparticles have non-spherical shapes. Ranjan et al. investigated different pillar shapes including I-shape, L-shape, inverted L-shape, and anvil shape for non-spherical bioparticle separations as seen in Fig. 12a. It is suggested that the pillar protrusions and grooves in I-shaped or L-shaped pillar will induce and maintain the rotational movement of non-spherical bioparticles including red blood cells and bacteria, which results in the displacement mode in DLD array [19]. Furthermore, Holm et al. [17] presented an easy-to-use, rapid, and accurate DLD detection platform for trypanosomes from the blood by combining several DLD arrays of different depths into a series configuration, which exploits the irregular shape of trypanosomes for separation as seen in Fig. 12b. In a recent study, Beech et al. designed a DLD array to separate human bacterial pathogens, Streptococcus pneumoniae, into different subpopulations based on the bacterial chain length, which is one of the known virulence factors to cause severe disease (Fig. 12c). The separation may enable more detailed research on the relationship between the different morphologies and chain lengths of bacteria with their virulence mechanisms [81].

#### Applications of Deformability-Based Separation

**Fig. 13:**
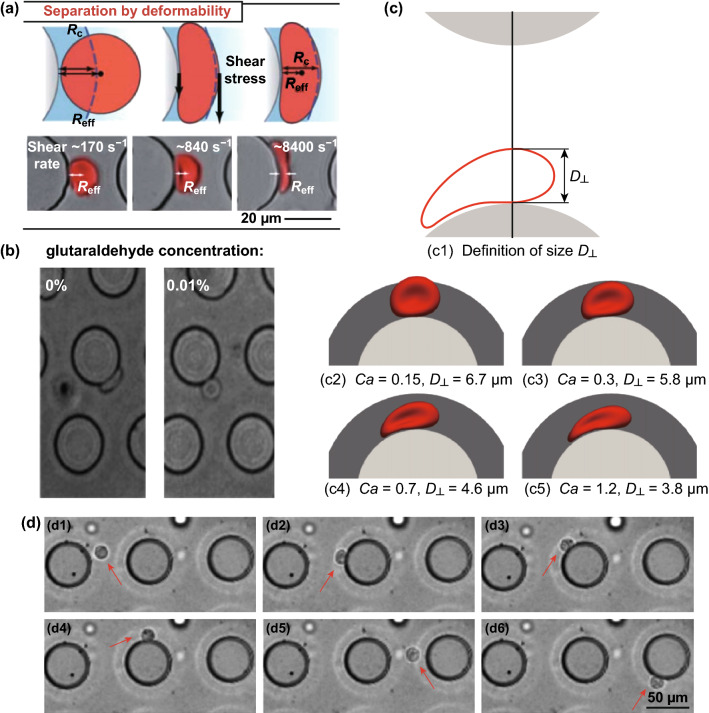
Applications of deformability-based separation. **a** The influence of shear rates on the effective radius of RBCs separation in DLD [15]. **b** The simulation study with different capillary numbers and stiffnesses of red blood cells [91]. **c** The effect of glutaraldehyde on RBC stiffness and the separation in DLD [82]. **d** The application of high flow rate to induce the deformation on MG-63 cells that leads to the zigzag trajectory in DLD (red arrow, from the left to the right) [16]. Figure panels reproduced from Ref. [15] with permission from the Royal Society of Chemistry, copyright 2012, Ref. [91] with permission from the AIP publishing, copyright 2014, Ref. [82] with permission from the Royal Society Publishing (CC BY 2014), Ref. [16] with permission from the Royal Society of Chemistry, copyright 2018

Most of the samples that are handled by DLD are biological samples, which are typically soft particles with deformable shapes, especially red blood cells. While the DLD model provides a high accuracy for predicting rigid spherical particle separation, the critical diameter of the deformable particles is difficult to be predicted by DLD empirical formula as the deformation of the particles leads to the smaller effective radius of the cells due to the flow shear as seen in Fig. 13a. Several simulation studies reported that the separation of deformable particles depends on the flow rate, fluid viscosity, and other intrinsic parameters such as the capillary number [43, 88–90].

Due to the change in the deformability of red blood cells induced by some diseases such as malaria which increases the cell stiffness, it is possible to separate and detect the diseased cells from the healthy cells. Kruger et al. [91] showed via three-dimensional immersed-boundary lattice Boltzmann simulations that the diseased (stiffer) RBCs have a higher displacement due to the less deformation on the pillar gap as seen in Fig. 13b. There were also experimental results that demonstrate the effect of cell stiffness and deformability on RBCs separation. With the use of glutaraldehyde (0–0.01%) to increase the stiffness of RBCs, it is observed that stiff RBCs show a higher migration angle compared to the healthy cells as seen in Fig. 13c [82]. It is also reported that the use of the sharp-edged pillar shapes including the triangle and diamond shape is able to improve the deformability-based separation [92]. In addition to the separation of red blood cells, the deformability-based separation has been demonstrated to improve the separation efficiency for circulating tumor cells and stem cells [16, 83]. Xavier et al. [16] applied the deformability parameter to improve the sorting of the human osteosarcoma cell lines MG-63 from HL-60 with the use of high flow rate during the separation process, which increases the purity from ~ 90 to 98% as seen in Fig. 13d. In another report, Liu et al. demonstrated an integrated DLD array combining two DLD modules, with the first step of two-mirrored DLD arrays for blood cells removal and the second step of DLD module with an increasing tilt angle for CTCs separation based on the size and deformability. This device has more than 90% capture yield, > 50% capture purity for the CTCs separation. Furthermore, the deformability analysis offers a biomechanical parameter to evaluate the metastatic potential of the CTCs [83].

#### Applications of Electrical Properties-Based Separation

DLD can be used for the separation of particles based on their electrical properties, including the dielectric properties and charges. The dielectric property of the particle can be employed for the separation parameter in DLD with the use of the electric field to generate the dielectrophoresis (DEP) force. In a non-uniform electric field, the particle with less polarizability than the surrounding medium results in a negative DEP, which pushes the particle toward the region of low electric fields. Hence, the DLD combined with the DEP force could separate particles based on the particle polarizability parameter [12, 72]. Although multiple researches have designed and investigated the influence of DEP on the particle/beads separation under different conditions, the application of the DLD-DEP on cell separations has not been thoroughly studied. Aghaamoo et al. [71] simulated the combination of DLD-DEP with different applied voltages, velocities, and geometrical parameters for separation of the similar-sized MDA-231 breast cancer cells with different WBC cell types. Another electrical property of the particle that can be employed for DLD separation parameter is the particle surface charge. Due to the dominance of electrostatic interaction effect in nanoregime DLD, the separation of particles with different surface groups and charges has been possible. It has been reported that the polystyrene (PS) beads with different surface charge groups of plain PS, PS-COOH, PS-NH_3_, as well as albumin-coated beads have different lateral displacement extents and apparent diameters in DLD [23].

### DLD for Particle Detection

**Fig. 14:**
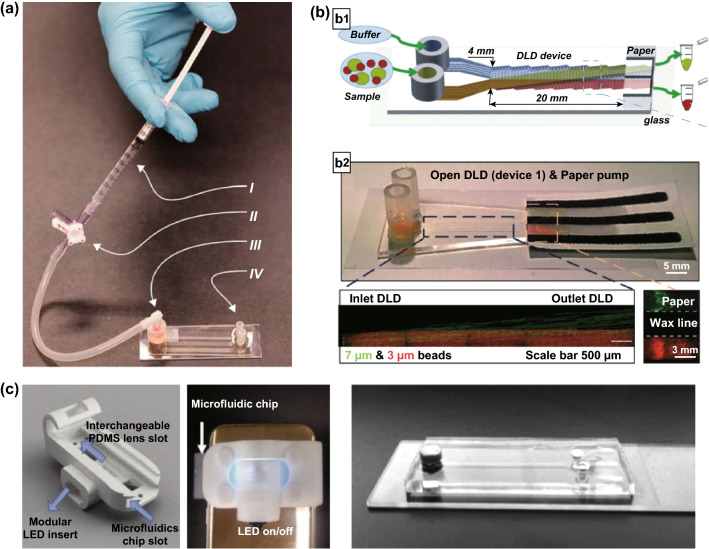
Simplification of DLD setups for particle separation. **a** The use of a simple disposable syringe-based pump to drive the fluid in microfluidic DLD [17].**b** The application of paper pump to drive the particle separation in an open DLD channel [69]. **c** A portable smartphone-based platform for moving beads detection in microfluidic DLD [93]. Figure panels reproduced from Ref. [17] with permission from the Royal Society of Chemistry (CC BY 2016), Ref. [69] with permission from the Royal Society of Chemistry (CC BY 2017), Ref. [93] with permission from Wiley, copyright 2018

DLD has been established and widely used as a label-free particle separation platform. Recently, a fluorescent label-free detection platform based on the DLD pillar array with a bead-based nanobioparticle coating method was reported. This method is performed by correlating the lateral shifts of the nanobioparticle-coated microbeads with the concentration of bioparticles bounded on the microbeads [23]. There are two different detection modes, which are the charge- and size-dominant detections as seen in Fig. 14. The charge-dominant detection exploits the electrostatic interaction between the particle and pillars for biomolecule detection. The coating of biomolecules such as proteins modulates the surface charge of the beads, which changes the migration angle and apparent diameter in DLD arrays due to the higher negative surface charge after coating. This label-free protein detection has been demonstrated to provide a limit of detection down to 10 ng mL^−1^ or 150 pM of albumin protein concentration. On the other hand, the size-dominant detection employs the size-based displacement from the coating of nanobioparticles such as vesicles or exosomes that have a larger size of more than 50 nm. This is because the coating of the nanobioparticles could increase the size of the 1-µm bead to the size range of 1.05 to 1.4 µm. The polymer vesicle detection in DLD has been demonstrated based on the difference in the lateral displacement position of the vesicle-coated microbeads as compared to the control beads with a limit of detection of 3.75 µg mL^−1^. This label-free detection method provides a fast, inexpensive, and real-time detection of nanobioparticles with only requiring a standard laboratory microscope for measuring the lateral displacement of the beads on the outlet channel.

## Challenges and Potential Solutions of DLD

DLD technology has been developed for more than a decade since its conception in 2004. Although DLD has been shown to be a versatile particle separation method with a high resolution for various applications, there are several limitations of using DLD, mainly due to the low throughput, pillar clogging issue, and bulky DLD experimental setup. These limitations have been continuously addressed to bring DLD as an ideal analytical tool for commercial applications.

### Low Separation Throughput

As DLD arrays use many pillar structures, the fluid resistance of the microfluidic DLD is comparatively higher compared to other microfluidics techniques, which limits the application of DLD for large sample volume separations. Several methods have been reported to improve the throughput rate. The use of sparse pillar arrays with only several pillar structures and the sieve-based lateral displacement is able to improve the throughput rate in DLD, but it requires an additional adjustment on the array design to balance the pressure to prevent the disruption of the particle separation [94–97]. Stacking and parallelization of microfluidic DLD devices to multiply the throughput rate have also been reported [37, 98–100]. For example, the combination of 15 parallel microfluidic DLD devices is able to process over 100 mL of blood in less than an hour [78]. Furthermore, an integrated parallel nano-DLD device that is comprised of 1024 devices on a single chip has been demonstrated for exosome isolation at the flow rate of up to 900 μL h^−1^ [79]. The fabrication of deep microfluidic DLD is also a way to improve the flow rate, but it depends on the aspect ratio of the pillar fabrication method. The increase in the channel width is also a possibility to improve the throughput rate, but it requires a longer microfluidic DLD length to provide the full lateral displacement of the particles for a large channel width. The use of a high flow rate to achieve faster particle separation can also improve the throughput rate, but with the caution of the maximum microfluidics channel integrity to avoid delamination of the channel, as well as the change in the critical diameter of the separation due to the streamline disruption at a high Reynolds number regime.

### Particles Adhesion and Clogging

Another challenge of DLD is the particle clogging issue due to the presence of multiple pillar arrays with the small gap between the pillars. The clogging could result in the inefficiency of the separation due to the disruption of the streamline around the clogged area that changes the critical diameter of the separation [42]. To prevent this clogging, the microfluidics surface is typically passivated with different surfactants including PEG, Pluronic, or Tween-20 prior to the use for separation applications. However, the clogging may still persist even after this surface treatment process. This clogging can be minimized using the DLD with a large lateral gap such as using the asymmetrical gap, triangular and optimized pillar shape. This is because these arrays can have a larger lateral gap to produce the same critical diameter size when the typical circular arrays are used. The optimized pillar shape provides the highest asymmetrical fluid flow between the gaps and has been proven to significantly reduce the particle clogging compared to the circular pillar shape. Another option that potentially could reduce the clogging in DLD is to use the liquid bridges as the stationary phase [101]. The use of filter pillar arrays on the inlet channel before the actual DLD arrays is also a common design practice to avoid the clogging from the unwanted particles that are larger than the DLD gap size [102]. In the blood fractionation application, the clogging commonly occurs due to the blood clot, especially for processing a large volume of blood with a high flow rate. Because of the clot, DLD blood fractionation is often limited to process 100 to 200 µL volume of blood. The inhibition of clot in microfluidic DLD for blood processing has been systematically studied, and the combination of 5 mM EDTA and 40 μM PPACK is recommended to inhibit the clot in DLD, which is proven to process 14 mL of blood at the velocity of 20 cm s^−1^ in less than 45 min without clotting [78]. Another solution to the clogging issue is to use the open-channel microfluidic DLD. This open structure enables the cleaning of the microfluidic structures by sonication and rinsing when the clogging occurs. The rinsing of the open-channel structure can achieve 30 × fewer trapped particles than the closed-channel DLD [69]. The bubble formation on the pillar arrays is also one of the issues in microfluidic DLD that can lead to the flow disruption and clogging. With the poor device priming, the bubbles can be formed and trapped inside the channel. Once the bubbles are trapped on the pillar arrays, they are difficult to be removed and may disrupt the particle separation process. Several device priming methods have been proposed to prevent the bubble formation in DLD. The application of surfactant solutions including 0.5–1% Pluronic and 1% Tween 20 (w/v) is able to wet the channel and purge the air bubbles in the arrays [82, 103]. To avoid the bubble formation in the nanoscale DLD array, the channel can be fully wetted through capillary action from the inlet holes, with the care of not to splash any liquid on the outlet port as it may lead to the bubble formation. Another method is to use the autoclave on the submerged chip in the beaker to enable the water to fill the channel without the bubble formation [20, 104].

### Complex and Bulky Experimental Setup

DLD has been demonstrated for separation of parasites from blood, diseased RBCs, bacteria, exosomes, etc. However, there is a gap between the experiments of microfluidic DLD in the laboratory with the application in the field. Although the DLD chip designs typically fit on the size of a microscope glass slide and can be mass-produced at a low cost, they still require expensive, bulky, and high power consumption pressure controls to pump the fluid, laboratory microscopes for imaging, and computer systems for data analysis. These requirements hinder the implementation of DLD outside the laboratory settings for point-of-care applications in a remote area. Therefore, the simplification of the DLD setup is required to create a portable, less power consumption, and low-cost solution for point-of-care diagnostics in the low-resource setting. Several researchers have addressed this limitation by developing a portable pump for microfluidic DLD. Holm et al. [17] developed a pressure generator based on the disposable syringe with a control valve to create a decompressed air, driving the fluid into a simple DLD with only one inlet to sort different particles such as leukocyte, RBCs, and trypanosomes as seen in Fig. 14a. Another portable pump based on the paper materials on an open DLD channel has also been reported to drive the separation of MCF-7 cells from RBCs as seen in Fig. 14b. Furthermore, the evaporation-induced pumping method has been demonstrated to drive the fluid in an open-top nanoarray [105]. The degas-driven pump in PDMS-based DLD device has also been reported to separate blood cells [106]. The simplification of microfluidic DLD experimental setup has also been demonstrated using a compact and low-cost smartphone-based platform to detect moving beads in the microfluidic DLD with PDMS lens, plug-and-play paper pump, and 3D printed smartphone dongle as seen in Fig. 14c [93].

## Conclusion and Perspective

DLD has been widely used for particle separation due to its low cost, simplicity, robustness, and precise manipulation of critical diameter to separate particles based on their size, shape, deformability, and charge. Since its conception, there are various modifications on DLD designs, including the pillar size, shape, gap size, the variation in channel width, and device layout to improve the separation efficiency and throughput. Several methods to tune the critical diameters have also been demonstrated for various particle separations. Furthermore, the electrostatic phenomena in DLD provide new opportunities for nanoparticle separation and nanobioparticle detection in a microfabricated array, while the true nanoscale DLD array has been reported for exosomes isolation and DNA enrichment with high efficiency. DLD has also been applied for the high-throughput particle separation with a mirrored, highly sparse, parallel, and asymmetrical gap design to support a large-volume sample processing. DLD technology is likely to be further explored for various medical applications including stem cells research and biomolecules (DNA, protein, exosomes) separation and detection. As the limitations of DLD such as throughput and clogging issue have been addressed, DLD is considered to be a mature technology that can be implemented for the real-world application. However, the industrial applications based on DLD separation are still in an early stage. Recently, microfluidic DLD has been fabricated with plastic materials that have the potential to be mass-produced. With plastic-based microfluidics, commercialization of the DLD technology has been initiated for T cells isolation from the leukapheresis sample for front-end cell therapy manufacturing. Similarly, the commercialization of nanoscale DLD requires the adoption of more affordable materials and manufacturing cost. DLD technology is predicted to evolve toward the compact point-of-care testing (POCT) device for medical applications. To accelerate the POCT applications, the integration of both particle separation and further downstream analysis including particle detection needs to be established. Furthermore, the simplification of the setup including the portable pump and detection systems needs to be further explored to achieve a compact and portable solution. With this outlook, the rapid and low-cost particle separation and detection using microfluidic DLD have tremendous potential impact for point-of-care diagnostics in the future.
